# Computer Simulation of Platelet Adhesion around Stent Struts in the Presence and Absence of Tissue Defects around Them

**DOI:** 10.1155/2021/8880988

**Published:** 2021-02-12

**Authors:** Yota Kawamura, Noriko Tamura, Shinichi Goto, Shinya Goto

**Affiliations:** ^1^Department of Medicine, Tokai University Hachioji Hospital, Hachioji, Japan; ^2^Department of Health and Nutrition, Faculty of Health Sciences, Niigata University of Health and Welfare, Niigata, Japan; ^3^Department of Medicine (Cardiology), Tokai University School of Medicine, Isehara, Japan

## Abstract

**Aim:**

To predict platelet accumulation around stent struts in the presence or absence of tissue defects around them.

**Methods:**

Computer simulations were performed using virtual platelets implementing the function of the three membrane proteins: glycoprotein (GP) Ib*α*, GPIIb/IIIa, and GPVI. These platelets were perfused around the stent struts implanted into the vessel wall in the presence or absence of tissue defects around them using within the simulation platform. The number of platelets that adhered around stent struts was calculated by solving the blood flow using Navier–Stokes equation along with the adhesion of membrane protein modeled within the platform.

**Results:**

Platelet accumulation around stent struts occurred mostly at the downstream region of the stent strut array. The majority of platelets adhered at the downstream of the first bend regardless of the tissue defect status. Platelet adhesion around stent struts occurred more rapidly in the presence of tissue defects.

**Conclusion:**

Computer simulation using virtual platelets suggested a higher rate of platelet adhesion in the presence of tissue defects around stent struts.

## 1. Introduction

Coronary stent implantation is a common procedure for treating patients with acute and chronic coronary artery diseases [1–3]. Indeed, stent implantation is effective for preventing acute phase coronary occlusion by restoration of normal anatomy [4]. Stent thrombosis occurring in the subacute phase is managed well with appropriate antiplatelet therapy [5]. Antiplatelet therapy helps to maintain the arterial lumen, and thus, it also facilitates maintenance/restoration of normal anatomy. Recently, drug-eluting stents (DES) using sirolimus [6, 7], paclitaxel [8, 9], or zotarolimus [10] have been shown to prevent restenosis by inhibiting smooth muscle growth [11]. Drug-eluting stents were proven to be effective in reducing the need for target vessel revascularization in patients at high risk for restenosis without a significant increase in the risk of myocardial infarction or death [12].

Due to the reduced risk of thrombotic complication with recent stents, the more recent recommended length for strong antiplatelet therapy using the combination of aspirin and P2Y_12_ inhibitors has been shortened [13–15]. In general, the risk of thrombotic events is no longer higher than the risk of serious bleeding complication caused by dual antiplatelet therapy several months after DES implantation [16]. Stent thrombosis in late term is rare but is an event that can lead to severe, negative patient outcomes including death [17, 18]. However, it is suggested that risk stratification based on various conditions can be helpful to identify patients who require extended antiplatelet therapy [19]. Risk stratification demonstrates that there is a higher risk of late phase stent thrombosis in patients with malapposition of stents [20–24]. Thus, it may be reasonable to consider longer DAPT duration for patients with stent malapposition. However, extending DAPT duration is associated with a higher risk of serious bleeding complication [25, 26]. Thus, further studies are needed to develop safer treatment strategies that specifically target patients with stent malapposition.

The mechanism of an increased risk of stent thrombosis in patients with stent malapposition is not understood [23, 27]. This lack of understanding creates a barrier for developing new treatment protocols. In patients with drug-eluting stents, stent malapposition was associated with tissue defects around stent struts due to delayed tissue repair [28]. This has been suggested to be the result of drugs inhibiting cell proliferation [26] and could be contributing to high thrombosis rate [22]. From these findings, the present hypothesis was that tissue defects around stents caused by malapposition are contributing to the high rate of thrombosis by increasing the rate of platelet adhesion to the vessel wall.

Recent advances in the calculation power of computer and information technology facilitates in silico analysis by constructing various complicated biological phenomena such as thrombus formation by solving basic physical/chemical equations [27, 29, 30]. Using this technology, computer simulation models of platelets adhesion under various blood flow conditions were developed [29]. In this study, these computer simulations are applied to examine the contribution of tissue defects around the stent struts on the rate of platelet adhesion.

## 2. Method

### 2.1. Virtual Platelet

Virtual platelets implementing the function of adhesion at site of endothelial injury, activation, and aggregation were used in this study. The details of virtual platelets have been published previously [29]. The function of three major platelet membrane proteins, namely, glycoprotein (GP) Ib*α*, GPIIb/IIIa, and GPVI, was integrated into the virtual platelet, computer simulation as in the Kelvin–Voigt model [29]. Virtual platelets adhere at the region of virtual endothelial injury mimicking the function of GPIb*α* interacting with the von Willebrand factor (VWF) and collagen fibrils exposed to the blood stream [29]. The outside-in signaling caused by stimulations of GPVI (by collagen) [31], GPIb*α* (by VWF) [31], and other chemical receptors on platelets (by various soluble agents such as adenosine 5'-diphosphate (ADP) [32], epinephrine [33], and thrombin [34]) were modeled by the activation of virtual platelet upon adhesion to the vessel wall. To model the firm adhesion caused by platelet activation, the binding constant was increased upon platelet adhesion on VWF/collagen as published previously [29]. The validity of the abovementioned virtual platelet model was confirmed by extended computer simulation implementing the local activation of coagulant cascade on activated platelets [30]. Biological validity of the virtual platelets used in this study was confirmed by experiments using human blood specimens and parallel plate flow chambers [31–36].

### 2.2. Solving Blood Flow around Stent Struts

Blood flow around stent struts were calculated by solving the discretized Navier–Stokes equation [27, 29, 30]. Briefly, the diameter of the target vessel was set to 3 mm. Blood flow was simulated as pulsatile flow with a linear change in blood flow from 0 to 22.5 mm/sec. The initial density of platelets in blood was set to 300 × 10^6^/ml and distributed uniformly. The Δ*t* for calculation was defined as 0.01 second.

**Figure 1:**
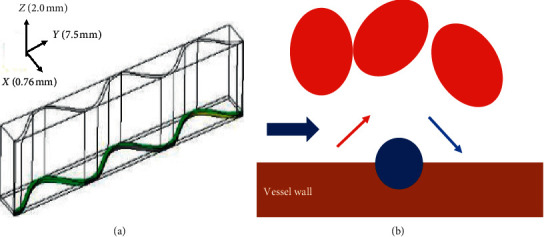
Shapes of stent strut implanted into vessels. (a) The shape of stent struts implanted into the vessel from diagonal view. Arrows indicate *x-y-z* coordinates. (b) This is a cross-sectional view of the implanted stent struts (blue). The initial blood flow and blood flow after passing stent struts (black arrow). Both are laminar flow. At the site of implanted stent strut, *z*-axis component of blood flow appears as blood flow turbulence induced by flow-obstacle interaction which determines local flow dynamics (red arrow). Since the overcoming flow collide to the heavier red blood cells (shown as red particle) present in the center of blood flow, *z*-axis direction of blood flow changed to go forward to the vessel wall following the physical law of action and reaction (green arrow). Platelets are not shown because platelet flows in similar fashion as medium. Initial blood flow has only *y*-axis components (*Vy*).

The stents are implanted as shown in Figure 1(a). Stent struts were implanted into the vessel wall as shown in Figure 1(b). The stent strut has a round shape with a diameter of 0.09144 mm. Half of the diameter of stent struts was buried in the vessel wall, but the other half was exposed to the blood stream. The initial direction of blood flow was set to be parallel to *y*-direction (*x-* and *z*-axis components were 0).

### 2.3. Tissue Defect around Stent Strut

**Figure 2:**
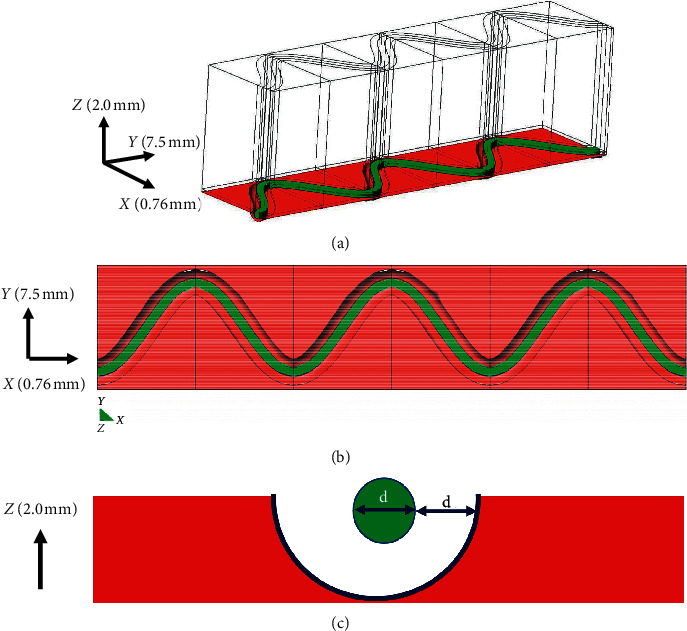
Tissue defect around stent strut. Virtual stent struts are implanted. The diagonal view of the area of interest corresponding to Figure 1(a), but in the presence of tissue defects around implanted stent struts. Arrows indicate *x-y-z* coordinates. B and C show the view from the top and the side. The thick green line represents the implanted stent strut (panels A, B, and C). The diameter of strut (d) in panel (C) is 0.09144 mm. The outer margin of physical (i.e., tissue) defect around stent struts is shown as blue line.

Tissue defects are organized around the stent struts as illustrated in Figure 2. The sizes of tissue defects were equal to the diameter of stent struts (Figure 2(c)).

### 2.4. Conditions for the Simulation

Virtual blood with the density of 1,000 kg/m^3^ and dynamic kinematic viscosity coefficient of 0.001004 m^2^/s was perfused for 30 seconds in the virtual vessel with the diameter of 3 mm and in the presence of stent struts with or without tissue defects. The number of virtual platelets adhering around stent struts was calculated with a high-performance computer equipped with an Intel® Xeon Phi^TM^ 7210 processor (Intel Corporation, Santa Clara, CA, USA). The distribution of platelet adhesion at 1-, 3-, and 10-second perfusion of virtual blood is shown in both conditions in the presence and absence of tissue defect around stents. Time-dependent changes in the number of platelets adhering to the area around stent struts were calculated. Calculated results are also shown as supplemental movies.

## 3. Results

### 3.1. Blood Flow toward the Vessel Wall Was Observed Downstream of Stent Strut

**Figure 3:**
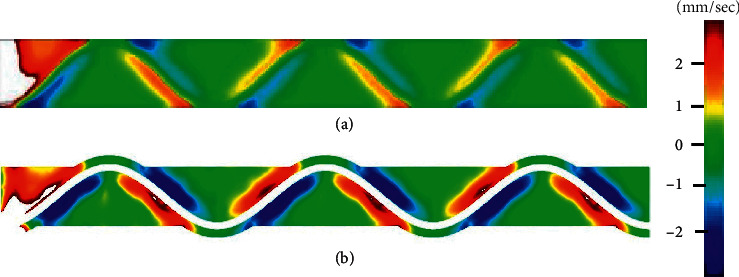
Distribution of the *Z*-axis direction of blood flow around stent strut. (a) Diagram of the distribution of the velocity of Vz on XY plane of the vessel wall implanted by stents in the absence of tissue defect. The velocity of Vz towards the vessel wall is shown as gradient blue, while the velocity towards the center of blood flow is shown as gradient red. (b) This image shows the distribution of *z*-axis direction of blood flow in the presence of tissue defect around stent struts.

Although the initial *z*-axis component of blood flow velocity was 0, flow in the *z*-axis direction was observed when the blood flow reached the stent struts. The distributions of *z*-axis components of blood flow around stents at maximum velocity (mm/sec) in the absence and presence of tissue defect are shown in Figures 3(a) and 3(b), respectively. *Z*-axis direction velocity toward the vessel wall was higher downstream than upstream of stent strut.

### 3.2. Platelet Adhesion Was Pronounced around the First Stent Struts Compared to the Second Strut

**Figure 4:**
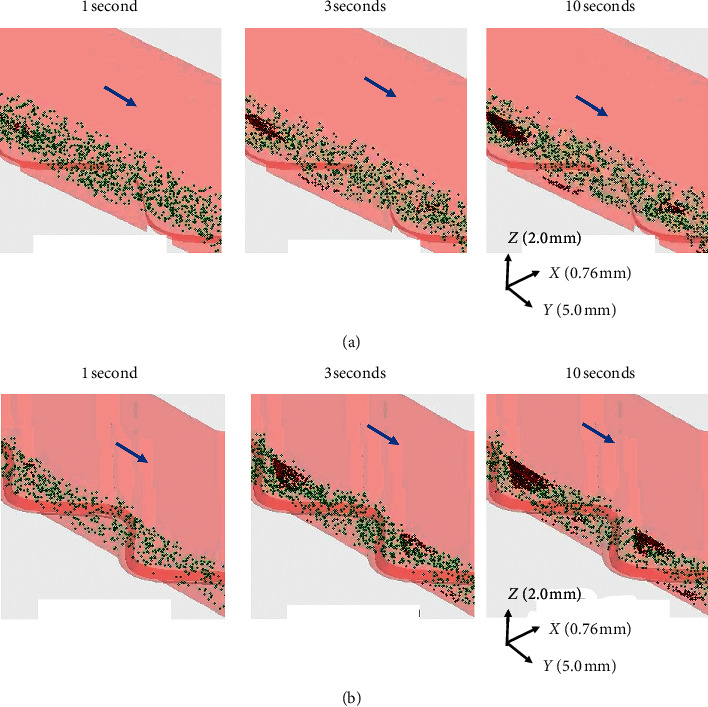
Distribution of platelet adhesions around stent struts in the presence and absence of tissue defect. (a) The distribution of adherent platelet in the absence of tissue defect around stent strut is shown at 1, 3, and 10 seconds after virtual blood perfusion without tissue defects. (b) In the presence of tissue defects, the corresponding results at 1, 3, and 10 seconds. Adhered platelets are shown as red particles. Blue arrows indicate the direction of blood flow in these 3-dimensional projection images. Arrows indicate *x-y-z* coordinates.

Figure 4 shows the snapshots of three-dimensional (3D) projection view delineating platelet adhesion around stents in the presence and absence of tissue defects. Almost no platelets adhered around the stents at 1 second both in the presence and absence of tissue defect. Adhered platelets increased in a time-dependent manner from 3 to 10 seconds. Despite similarly higher velocity of *z*-axis blood flow toward the vessel wall in the downstream of stent struts (Figure 3), more platelet adhesions were observed at downstream of the first stent strut as compared to the second at 3 seconds. The difference was larger in the absence of tissue defects (Figure 4(a)) as compared to the condition with tissue defects (Figure 4(b)). At 10 seconds, more platelet adhesion was observed in the presence of tissue defects compared with no tissue defects. Although the platelet adhesion was increased with tissue defects in downstream of both the first and second stent struts, the difference was larger around the second stent strut than the first. Full movie demonstrating the time-dependent changes in the number of platelets adhered around stent strut in the presence and absence of tissue defect is provided as supplementary materials (Movies A and B).

### 3.3. Platelet Adhesion Was Increased with the Presence of Tissue Defect

**Figure 5:**
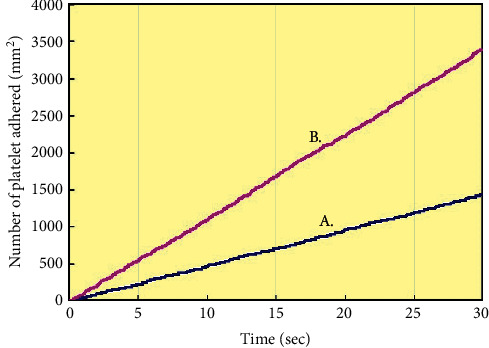
Number of platelet adhesions around stent struts in the presence and absence of tissue defects. Time-dependent changes in the number of platelets adhered in the area shown on Figure 4 in the absence (A blue line) and presence (B red line) of tissue defects around stent struts.

Figure 5 shows the time-dependent increase in the number of platelets adhered in the simulation area shown as XY plane in Figure 3 from 0 to 30 seconds. Indeed, the number of adhered platelets was higher in the presence of tissue defects than that in their absence. At 30 seconds, it reached to 490/mm^2^ in the absence of tissue defects and reached 3420/mm^2^ in its presence.

## 4. Discussion

Supporting initial hypothesis, the computer simulation suggested higher rate of platelet adhesion around stent struts implanted into the vessel wall in the presence of tissue defects around them as compared to absence tissue defects. This difference is likely due to the changes in the distribution of *z*-axis component of platelet motions induced by the presence of stent struts because the *z*-axis direction velocity of blood flow toward the vessel wall was apparently higher downstream than upstream of stent struts. This was further supported by the finding that the *z*-axis direction velocity at downstream of stent strut in the presence of tissue defects was higher as compared to that without defects.

The results of the present research suggest that actual platelet adhesion is not fully determined by the *z*-axis velocity of blood flow. Indeed, apparently, more platelet adhesion was observed downstream of the first stent strut than the second strut at 3 and 10 seconds of blood perfusion regardless of the presence of tissue defects. The reason for this difference is not fully understood. Reduced platelet density around the second stent strut caused by the consumption of platelet by adhesion around the first stent strut is one potential explanation. However, platelet adhesion was increased with tissue defects regardless of the locations and thus supports the conclusion that tissue defects may be an important contributor for higher rate of stent thrombosis in patients with late malapposition.

Potential factors influencing the risk of late stent thrombosis are summarized in Table 1 [37–39]. General factors such as thrombogenicities exerted by platelet reactivity are managed by the appropriate use of dual antiplatelet therapy [40]. Local factors such as the thrombogenicity of the stent struts were also improved by using less thrombogenic materials and shapes [36]. A critical issue, especially for the late stent thrombosis with the use of drug-eluting stents, is the malapposition [20]. Stent malapposition or incomplete stent apposition is a morphological description defined by the lack of contact between at least one stent strut and the underlying intimal surface of the arterial wall in a segment not overlying a side branch. Clinical observations suggest a higher prevalence of tissue defects around stents causing late stent malapposition in patients experiencing late stent thrombosis [23, 24, 26, 41, 42]. However, it is important to note that the prevalence of stent malapposition differs substantially with the use of different diagnostic devices and studies. Even with the use of intravascular ultrasound (IVUS), the prevalence of late stent-malposition ranges significantly between studies from the lowest of 0% at 8 months [43] to 25% at 9 months [44]. Other studies using optical coherence tomography (OCT) report high prevalence of acute stent malapposition in 62% of the lesions treated, and 31% of them remained as malappositions at 6 months [42]. New onset of late acquired malapposition was observed in 15% of the lesions [42]. These reports suggest that imaging devices cannot completely eliminate stent malappositions.

In general, late stent malapposition is recognized as a risk factor for stent thrombosis. But, a recent report by Im et al. also demonstrated that none of the 351 patients experienced clinical stent thrombosis within 8.6 ± 10.3 months of follow-up. Meta-analysis showed higher risk of late malapposition as compared to the bare metal stent with an odds ratio of 2.49 (CI 95% 1.15–5.35) [26]. Thus, stent malapposition is one of the risk factors for stent thrombosis, but the majority of late acquired stent malapposition remains asymptomatic. The results of the present investigation show a higher rate of platelet adhesion around stent struts in the presence of tissue defects in comparison with its absence. These results do not contradict clinical findings because the accumulation of platelets is one of the triggers for thrombus formation, but contributions from other factors such as local activation of coagulant factor is necessary to cause symptomatic coronary thrombosis [45].

Higher rate of platelet accumulation downstream of stent struts has been previously documented with human blood and flow chamber experiments [36]. This computer simulation finding is in good agreement with these previous biological, experimental findings. Detailed clinical observations with the use of OCT in patients with very late stent thrombosis revealed the presence of in-stent neointimal rupture in approximately 70% of patients [46]. The rate of neointimal rupture was higher than the prevalence of stent malapposition of 42% [46]. The contributing role of platelets in neointimal plaque formation remains to be elucidated [47, 48]. The higher rate of platelet accumulation shown here may suggest a contributing role of accumulated platelets for neointimal formation and future rupture.

### 4.1. Limitations

This study has several limitations as listed below.

- Predictive calculation in this simulation was conducted only for 30 seconds. It is likely that longer times are required for the process of stent thrombosis to be complete. Thus, the present results should be interpreted as predictive of only the initial part of the clinical phenomena, which might not necessarily result in clinically significant events such as stent thrombosis.
- The clinical thrombosis often appears as sudden cardiac death or ST-elevation myocardial infarction, which is likely to be caused by arterial occlusive thrombi including both platelet and fibrin [3, 49]. This simulation only predicted early platelet accumulation but did not include the process of coagulation cascade leading to fibrin formation.
- This simulation only included tissue defect around stent struts as a parameter but not others such as old age and renal dysfunction. Personalized prediction inclusive of these factors on the influence of stent thrombosis achieved may be understood by future studies.
- The interaction between platelets and erythrocytes should play a role for determining the motion of platelets. This factor was not included in the current analysis. Future studies examining this aspect are now being designed in this laboratory.

### 4.2. Fundamental Strengths of Simulation Calculation Are the Following

The numbers of platelets accumulated around stent could be quantitatively calculated from the basic physical principals.

## 5. Conclusion

In conclusion, higher rate of platelet adhesion around stent struts in the presence of tissue defects as compared with its absence is shown in this computer simulation study. This study results support the notion that higher rate of platelet adhesion in the presence of tissue defects is a risk factor for stent thrombosis. Computer simulation provides insight into the higher risk of stent thrombosis in patients with stent malapposition.

## Figures and Tables

**Table 1:** Factors influencing the risk of stent thrombosis.

General factors	Reasons for stenting (acute MI or chronic ones)
	General thrombogenicity including platelet responsibility
	Risk factor such as diabetes mellitus
	Percutaneous coronary intervention

Local factors	Shape and length of stent strut
	Components of atheroma
	Polymer and coated drugs
	Stent malapposition

## Data Availability

The results of this study are generated by simulation calculation.

## Abbreviations

DES: Drug-eluting stent
GP: Glycoprotein
IVUS: Intravascular ultrasound
OCT: Optical coherence tomography
PCI: Percutaneous coronary intervention
VWF: von Willebrand factor.

## Supplementary Materials

Supplementary Materials Movies demonstrating the platelet adhesion in the presence and absence of tissue defect. Movie A and movie B demonstrate the platelet adhesion in the absence and presence of tissue defect around stent struts, respectively. The size and direction of each image is the same as Figure 4.
